# First‐trimester Placental Ultrasound (FirstPLUS) study: prediction of fetal growth restriction using OxNNet‐derived first‐trimester placental volume

**DOI:** 10.1002/uog.70146

**Published:** 2025-12-06

**Authors:** S. Mathewlynn, L. Nicolatino Starck, D. Wright, Y. Yin, M. Soltaninejad, K. H. Nicolaides, A. Syngelaki, A. Galán Contreras, S. Bigiotti, E.‐M. Woess, M. Swinburne, S. Collins

**Affiliations:** ^1^ Nuffield Department of Women's and Reproductive Health University of Oxford Oxford UK; ^2^ Oxford University Hospitals NHS Foundation Trust Oxford UK; ^3^ Fetal Medicine Research Institute, King's College Hospital London UK; ^4^ Institute of Health Research University of Exeter Exeter UK; ^5^ University of Bristol Bristol UK; ^6^ Birmingham Women and Children's NHS Foundation Trust Birmingham UK

**Keywords:** crown–rump length, first trimester, gestational age, neural network (computer), placenta, reference values, three‐dimensional ultrasonography, volume measurement

## Abstract

**Objectives:**

To develop predictive models for fetal growth restriction (FGR) with and without the inclusion of OxNNet‐derived first‐trimester placental volume (FTPV), thereby evaluating the contribution of FTPV to these models and the extent to which FTPV percentile is associated with subsequent FGR.

**Methods:**

This study utilized data from the First‐trimester Placental Ultrasound (FirstPLUS) study, a longitudinal observational cohort study conducted at King's College Hospital NHS Foundation Trust, London, UK, between March and November 2022. Participants underwent routine ultrasound assessment between 11 + 2 and 14 + 1 weeks' gestation, in addition to three‐dimensional placental sonography. The OxNNet toolkit was used for automated placental segmentation and volume calculation. Multivariable logistic regression models were developed to predict FGR, incorporating maternal factors, first‐trimester biomarkers (serum pregnancy‐associated plasma protein‐A, mean arterial blood pressure and uterine artery pulsatility index) and FTPV.

**Results:**

The final cohort comprised 3500 pregnancies, of which 250 (7.1%) developed FGR. Low FTPV was found to be a risk factor for FGR, with an odds ratio of 1.736 (95% CI, 1.499–2.015) per unit decrease in FTPV *Z*‐score. Incorporating FTPV into the predictive model based on maternal factors and biomarkers significantly increased the area under the receiver‐operating‐characteristics curve (AUC) for predicting all cases of FGR, from 0.78 (95% CI, 0.75–0.81) to 0.79 (95% CI, 0.76–0.82) (*P* = 0.005). Subgroup analysis of normotensive and hypertensive cases demonstrated a statistically significant effect size for the prediction of FGR by FTPV *Z*‐score in both groups. The addition of FTPV to the model based on maternal factors and biomarkers for the prediction of normotensive FGR increased the AUC from 0.77 (95% CI, 0.74–0.80) to 0.78 (95% CI, 0.75–0.81) (*P* = 0.01). For preterm FGR, the AUC was 0.85 (95% CI, 0.78–0.92) with FTPV and 0.85 (95% CI, 0.79–0.92) without (*P* = 0.93); the absence of a significant difference may be due to a lack of power.

**Conclusions:**

FTPV *Z*‐score is a predictor of FGR. Integrating FTPV into predictive models significantly enhanced the discriminative ability for all cases of FGR, as well as for the subgroup of normotensive FGR. © 2025 The Author(s). *Ultrasound in Obstetrics & Gynecology* published by John Wiley & Sons Ltd on behalf of International Society of Ultrasound in Obstetrics and Gynecology.

## INTRODUCTION

Placental insufficiency resulting in fetal growth restriction (FGR) is a significant contributor to perinatal morbidity and mortality, and there are long‐term cardiovascular and metabolic consequences associated with being small‐for‐gestational age (SGA)^1^, ^2^, ^3^, ^4^, ^5^, ^6^, ^7^.

Antenatally, FGR can be identified using fetal biometry and Doppler velocimetry, and a Delphi consensus‐based diagnostic definition of FGR is available^8^. Once FGR is suspected, various national guidelines provide assistance in managing affected pregnancies, weighing the risks of iatrogenic birth at a given gestational age against those of continuing the pregnancy^9^. Appropriate management depends on the timely diagnosis of FGR, and it is therefore critical that high‐risk cases are identified early in pregnancy to facilitate scheduling of antenatal care and ultrasound examination. As such, novel first‐trimester biomarkers that may improve the prediction of FGR are of interest.

There is a well‐established correlation between placental and fetal (or neonatal) weight, and mounting evidence suggests a relationship between first‐trimester placental volume (FTPV), assessed using three‐dimensional (3D) ultrasound, and subsequent fetal growth^10^, ^11^, ^12^, ^13^, ^14^. Previously, the process of manual or semi‐automated placental segmentation was too time‐consuming or unreliable for FTPV to be considered for population‐level screening. However, recent advancements in artificial intelligence (AI) present an innovative solution through the OxNNet toolkit^15^. This software, the development of which has been described previously, utilizes a fully convolutional neural network to enable the complete automation of placental segmentation and volume calculation^16^, ^17^. With the use of OxNNet, the incorporation of FTPV into population‐level risk assessment for FGR becomes a viable prospect.

This study aimed to develop predictive models for FGR with and without the inclusion of OxNNet‐derived FTPV, thereby evaluating the contribution of FTPV to these models and the extent to which FTPV percentile is associated with subsequent FGR.

## METHODS

### Study design and setting

This research utilized data from the First‐trimester Placental Ultrasound (FirstPLUS) study, a longitudinal observational cohort study involving pregnant individuals undergoing first‐trimester screening at the Harris Birthright Research Centre for Fetal Medicine, King's College Hospital NHS Foundation Trust, London, UK, between March and November 2022. Participants were recruited during their routine ultrasound assessment conducted between 11 + 2 and 14 + 1 weeks' gestation. In addition to routine care, 3D ultrasound imaging of the placenta was performed. Following the single study visit, participants continued with standard maternity care, and data on subsequent pregnancy outcomes were retrieved from electronic patient records. This study adhered to the Strengthening the Reporting of Observational Studies in Epidemiology (STROBE) guidelines^18^.

The West Midlands–Solihull Research Ethics Committee granted ethical approval for the FirstPLUS study on 8 March 2022 (reference 22/WM/0039). Participants provided written informed consent prior to participation and could withdraw from the study at any time.

### Study population

Individuals attending for the first‐trimester combined test were approached. Those who agreed to participate and provided informed consent were included if they met the following criteria: singleton pregnancy; age ≥ 18 years; presenting for the first‐trimester combined screening test between 11 + 2 and 14 + 1 weeks' gestation; able to understand written or verbal English (or able to access appropriate methods of translation); and not deemed to be at risk, under stress, or limited in their ability to participate in the study activities in the opinion of the investigators. Cases were excluded if any of the following criteria were met: non‐viable pregnancy or uncertain viability (no detectable heartbeat); multiple pregnancy (more than one viable fetus) identified at the time of the scan; pregnancy with a major defect identified at the time of the scan; pregnancy found subsequently to be chromosomally abnormal on pre‐ or postnatal testing; or pregnancy for which the combined test could not be completed (e.g. it was not possible to obtain a nuchal translucency measurement). Terminations of pregnancy and miscarriages were also excluded, as were those cases that were lost to follow‐up or had incomplete outcome data.

### Definitions and reference values

FGR was identified as either birth weight < 3^rd^ percentile or birth weight < 10^th^ percentile accompanied by Doppler abnormality at the time of the final ultrasound scan. Doppler abnormality was specified as an umbilical artery pulsatility index (PI) > 95^th^ percentile or a cerebroplacental ratio < 5^th^ percentile at the final ultrasound examination before birth, based on the reference ranges of Ciobanu *et al*.^19^. Birth‐weight percentiles were determined using the population birth‐weight charts developed by the Fetal Medicine Foundation (FMF), as outlined by Nicolaides *et al*.^20^. SGA was defined as birth weight < 10^th^ percentile. FTPV *Z*‐scores and percentiles were calculated based on crown–rump length (CRL) as per the method described by Mathewlynn *et al*.^21^, using the provided R models, which were constructed using the lambda‐mu‐sigma approach^21^. Multiples of the median (MoM) values for pregnancy‐associated plasma protein‐A (PAPP‐A), mean arterial pressure and uterine artery (UtA)‐PI were calculated and modeled against gestational age in days, adjusted for maternal ethnicity, weight and smoking status, as appropriate.

Preterm birth was defined as delivery before 37 + 0 weeks, while preterm FGR was defined as preterm birth occurring in conjunction with the criteria for FGR outlined above. Pre‐eclampsia (PE) and gestational hypertension were defined according to the guideline of the American College of Obstetricians and Gynecologists (ACOG)^22^. Normotensive FGR was defined as cases meeting the criteria for FGR in the absence of gestational hypertension or PE.

Gestational diabetes mellitus was diagnosed via an oral glucose tolerance test, following the timing and diagnostic thresholds set by the National Institute for Health and Care Excellence (NICE)^23^. Stillbirth was defined as birth with no signs of life ≥ 24 + 0 weeks. Miscarriage was defined as ultrasound‐confirmed *in‐utero* fetal demise, or birth without signs of life ≤ 23 + 6 weeks.

### Ultrasound assessment

Ultrasound scans were conducted using GE Voluson™ E6 or E8 ultrasound machines (GE Healthcare, Zipf, Austria), equipped with a RAB4‐8‐D 3D/four‐dimensional (4D) curved‐array abdominal transducer (4–8.5 MHz). These procedures were carried out by FMF fellows who were adequately trained in the technique of 3D‐FTPV acquisition. The ultrasound machines were preconfigured with the appropriate settings, which were stored as a preset mode to ensure uniformity across all scans (detailed settings are provided in Table S1). Viability of the pregnancy was verified, and CRL and nuchal translucency measurements were obtained in accordance with standard protocols.

Pregnancy dates were based on CRL measurement^24^, except in cases of assisted reproduction, for which the date of embryo transfer was used.

The placental ultrasound scan involved first identifying the ideal probe placement for 3D imaging, typically near the center of the placenta. Grayscale gain settings were adjusted to clearly distinguish the placenta from the adjacent myometrium. A static grayscale volume was captured and reviewed to ensure that it included the entire placenta. If any part was missing or was in shadow, the angle was adjusted or the probe was repositioned, and the capture was repeated.

### Image processing and quality control

Images were obtained directly from the ultrasound machines as .vol files without wavelet compression and were analyzed retrospectively by the Placental Imaging Research Group, Nuffield Department of Women's and Reproductive Health, University of Oxford, Oxford, UK. The placenta was segmented and the placental volume calculated using the OxNNet toolkit, as described previously^16^, ^17^.

Before placental segmentation, trained research sonographers retrospectively reviewed all images to ensure complete placental capture. Images were excluded if the placenta was incomplete, incorrect machine settings had been used or the image quality was too poor to delineate the placenta. Power Doppler was used to facilitate the measurement of fractional moving blood volume (the details of which are beyond the scope of this paper), and cases were excluded if there was excessive power Doppler flash artifact (more than five large flashes).

In a subsequent round of quality control, segmentation of both the placenta and amniotic fluid was conducted, utilizing both the current version of OxNNet and the original OxNNet prototype. The performance of each method was evaluated by comparing the segmentations using the Dice similarity coefficient. Cases exhibiting poor agreement were reviewed manually by S.M. and M.So., and those in which the placental contour could not be determined visually (for example, owing to very poor image quality) were excluded from the analysis. Images were flagged for review based on three criteria: agreement on placental segmentation < 60%, irrespective of agreement on amniotic fluid segmentation; agreement on placental segmentation < 75% combined with agreement on amniotic fluid segmentation < 40%; and agreement on amniotic fluid segmentation < 40%, irrespective of agreement on placental segmentation.

#### OxNNet versioning and failure modes

OxNNet was built on previously published methods for multiclass segmentation using native implementations^16^. To support reproducibility and real‐world feasibility, the following software environment was used: Python 3.7 (https://www.python.org/), TensorFlow^25^ 2.7.0 (https://www.tensorflow.org/), NumPy^26^ 1.21.4 (https://numpy.org/) and SciPy^27^ 1.7.3 (https://scipy.org/). Processing takes approximately 5 s per scan. Failures typically occur when the original ultrasound data files are corrupted or saved in the wrong format (e.g. incomplete or damaged headers), which prevents successful conversion to NIfTI. If conversion fails, processing cannot proceed. If the multiclass segmentation algorithm fails to detect the placenta, amniotic fluid or fetus, an evaluation of placental volume will not be returned.

### Statistical analysis

Statistical analysis was performed using *R* version 4.3.1 and RStudio version 2024.09.0 + 375 (R Foundation for Statistical Computing Platform, Vienna, Austria).

#### Sample size estimation

The target sample size for the FirstPLUS study was determined using the method of Riley *et al*.^28^ to ensure a sufficient number of cases for constructing multivariable models. Assuming a rare outcome (2% incidence), 2171 cases would be adequate for a model with 20 parameters and a C‐statistic of 0.9. For a C‐statistic of 0.8, 2992 cases would be needed. Fewer cases would be required if fewer parameters were used and for outcomes with higher prevalence. To account for attrition and exclusions, a recruitment target of 4000 cases was pragmatically chosen.

#### Group comparisons

The characteristics of the FGR and non‐FGR groups were summarized as median (interquartile range (IQR)) for continuous variables and *n* (%) for categorical variables. Statistical comparisons were conducted using the Wilcoxon rank‐sum test for continuous variables and the chi‐square test for categorical variables. In cases for which the count was less than five, Fisher's exact test was used instead. Significance was set at *P* < 0.05.

#### Assessment of multicollinearity

Before developing multivariable logistic regression models, we assessed multicollinearity among predictor variables to ensure the stability and interpretability of the model estimates. We generated a comprehensive correlation matrix using a combination of Pearson, polychoric and polyserial correlations, depending on the type of variable. Pearson correlation was used for continuous–continuous variable pairs, polychoric correlation for categorical–categorical variable pairs and polyserial correlation for continuous–categorical pairs. Handling of previous pregnancy data is described below. Pairwise associations between variables were visualized using a heatmap, which helped to identify any highly correlated predictors (i.e. |r| > 0.8) that could compromise the models.

#### Development of models with and without FTPV

To evaluate the potential of FTPV as a predictor of FGR, we conducted a series of univariable and multivariable binomial logistic regression analyses using the glm function in base R with FGR as the binary outcome. Crude and adjusted odds ratios (OR) were calculated, and 95% CIs were estimated using the profile likelihood method. Multivariable models included maternal factors, first‐trimester biomarkers or both. All MoM values were log_10_ transformed. In each case, two models were developed, one including FTPV as a predictor and one excluding it, resulting in six models for the prediction of FGR. This process was repeated for the binary outcomes of preterm FGR and normotensive FGR, resulting in 18 models in total. Details of these models are presented in Appendix S1, and an R file containing the models is provided in Appendix S2.

#### Handling of previous pregnancy data

The inclusion of outcome data pertaining to previous pregnancies presents a challenge for the construction of the correlation matrix and predictive models because of the lack of data in nulliparous women. Interaction terms were employed to allow the inclusion of previous pregnancy data, with binary‐coded pregnancy outcome (previous PE, SGA in last pregnancy, previous PE plus SGA in last pregnancy, and no history of PE or SGA) included in combination with last birth‐weight percentile and interpregnancy interval (resulting in two sets of four mutually exclusive variables).

#### Evaluation of relationship between FTPV and FGR

To investigate the association between FTPV and FGR, we performed a logistic regression analysis with FTPV decile (derived from the *Z*‐score) as the predictor, and plotted both estimated effect size and odds with 95% CIs. To assess whether the relationship between FTPV *Z*‐score and FGR followed a linear trend on the logit scale, the odds of FGR (with 95% CI) were plotted against the mean *Z*‐score of the corresponding FTPV decile group. ORs were calculated directly rather than relative to a reference category.

To compare the risk between extreme percentiles of FTPV *Z*‐score, a further logistic regression model was used to assess the OR for the 90^th^ percentile relative to that for the 10^th^ percentile. The OR was calculated as the model coefficient multiplied by the corresponding percentile difference.

#### Comparison of models

Receiver‐operating‐characteristics (ROC) curves were constructed for each model to assess discriminatory performance in predicting FGR. The area under the ROC curve (AUC) with 95% CI was calculated, with higher AUC values suggestive of better discriminatory performance. For each model, sensitivity was calculated for a 5%, 10% and 20% false‐positive rate.

To compare the models with and those without FTPV as a predictor, the DeLong test was applied using the roc.test function within the pROC package, which accounts for correlation between ROC curves derived from the same population.

#### Subgroup analysis

Univariable logistic regression analysis was conducted to evaluate the prediction by FTPV *Z*‐score of FGR in normotensive and hypertensive cases. ORs were presented in the form of a forest plot.

## RESULTS

**Figure 1:**
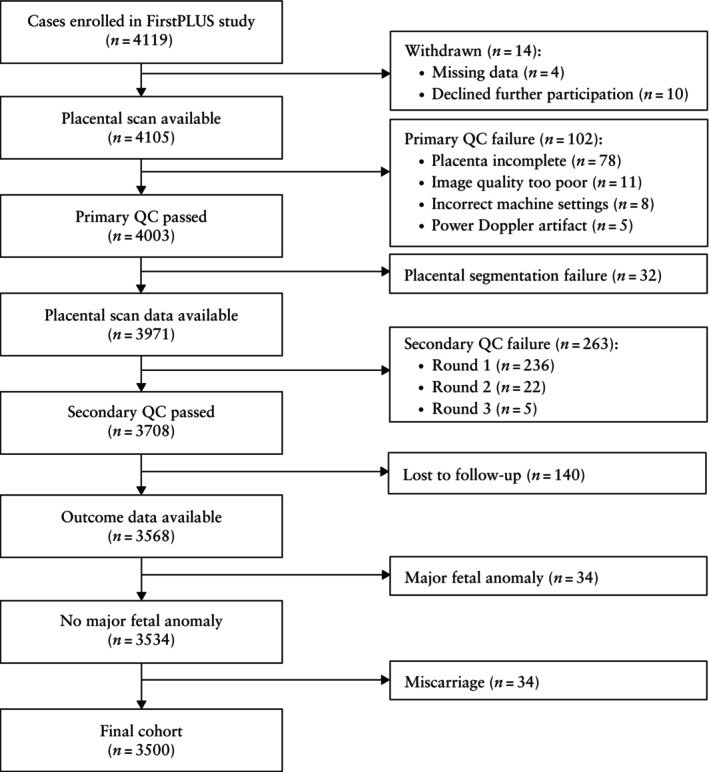
Flowchart summarizing selection of study cohort. QC, quality control.

After exclusions, the final study cohort comprised 3500 cases (Figure 1), of which 250 (7.1%) developed FGR. The characteristics of the final cohort are summarized in Table 1. Among the included cases, no data were missing, with the exception of neonatal sex, which could not be established in three cases.

**Table 1:** Maternal and pregnancy characteristics of study population, according to presence or absence of fetal growth restriction (FGR)

Characteristic	Non‐FGR (*n* = 3250)	FGR (*n* = 250)	*P*
Maternal age (years)	33.6 (30.5–36.4)	33.6 (29.8–36.6)	0.990
Maternal weight (kg)	67.2 (60.0–77.0)	64.0 (57.3–73.2)	< 0.001
Maternal height (cm)	166 (162–170)	163 (160–168)	< 0.001
Body mass index (kg/m^2^)	24.3 (21.8–27.9)	24.2 (21.4–26.9)	0.222
Ethnicity			< 0.001
Black	407 (12.52)	45 (18.00)	
East Asian	73 (2.25)	9 (3.60)	
South Asian	224 (6.89)	39 (15.60)	
White	2413 (74.25)	143 (57.20)	
Mixed race	133 (4.09)	14 (5.60)	
*In‐vitro* fertilization	245 (7.54)	18 (7.20)	0.943
Cigarette smoker at presentation	57 (1.75)	14 (5.60)	< 0.001
Family history of PE	94 (2.89)	8 (3.20)	0.933
Parity			< 0.001
Nulliparous	1560 (48.00)	149 (59.60)	
Parous, previous PE and last BW SGA	17 (0.52)	8 (3.20)	
Parous, previous PE only	60 (1.85)	2 (0.80)	
Parous, last BW SGA only	233 (7.17)	40 (16.00)	
Parous, neither previous PE nor last BW SGA	1380 (42.46)	51 (20.40)	
BW percentile in last pregnancy	43.7 (18.3–69.0)	12.0 (3.6–32.3)	< 0.001
History of stillbirth	0 (0)	0 (0)	NA
Interpregnancy interval (years)	2.48 (1.57–4.19)	3.66 (1.88–6.24)	0.002
Chronic hypertension	22 (0.68)	8 (3.20)	< 0.001
Pre‐existing diabetes mellitus			0.735
Type 1	23 (0.71)	2 (0.80)	
Type 2	22 (0.68)	2 (0.80)	
SLE/APS	11 (0.34)	4 (1.60)	0.018
PAPP‐A MoM	0.79 (0.55–1.11)	0.59 (0.41–0.85)	< 0.001
MAP MoM	1.00 (0.95–1.06)	1.01 (0.96–1.06)	0.024
UtA‐PI MoM	1.00 (0.81–1.24)	1.16 (0.92–1.44)	< 0.001
FTPV *Z*‐score	−0.01 (−0.06 to 0.61)	−0.52 (−1.01 to 0.06)	< 0.001
Gestational diabetes mellitus	325 (10.00)	33 (13.20)	< 0.001
Gestational hypertension	74 (2.28)	2 (0.80)	0.172
PE	79 (2.43)	25 (10.00)	< 0.001
Stillbirth	7 (0.22)	3 (1.20)	0.029
Neonatal death	2 (0.06)	0 (0)	NA
Neonatal sex			< 0.001
Female	1569 (48.28)	165 (66.00)	
Male	1679 (51.66)	84 (33.60)	
Not known	2 (0.06)	1 (0.40)	
Gestational age at birth (weeks)	39 + 4 (38 + 6 to 40 + 4)	38 + 3 (37 + 2 to 39 + 3)	< 0.001
Birth < 37 + 0 weeks' gestation	123 (3.78)	39 (15.60)	< 0.001
BW (g)	3405 (3145–3710)	2515 (2306–2684)	< 0.001
BW percentile	52.0 (30.0–75.2)	2.2 (0.8–5.4)	< 0.001
BW < 10^th^ percentile	153 (4.71)	250 (100)	NA

Data are given as median (interquartile range) or *n* (%). APS, antiphospholipid syndrome; BW, birth weight; FTPV, first‐trimester placental volume; MAP, mean arterial pressure; MoM, multiples of the median; NA, not applicable; PAPP‐A, pregnancy‐associated plasma protein‐A; PE, pre‐eclampsia; SGA, small‐for‐gestational age (defined as BW < 10^th^ percentile); SLE, systemic lupus erythematosus; UtA‐PI, uterine artery pulsatility index.

### Baseline characteristics

Statistically significant differences between the outcome groups were observed with respect to several characteristics. The FGR group demonstrated lower median maternal weight compared with the non‐FGR group (64.0 (IQR, 57.3–73.2) kg *vs* 67.2 (IQR, 60.0–77.0) kg; *P* < 0.001), as well as lower median maternal height (163 (IQR, 160–168) cm *vs* 166 (IQR, 162–170) cm; *P* < 0.001). The FGR group included a higher proportion of Black participants (45/250 (18.0%)) and South Asian participants (39/250 (15.6%)) compared with the non‐FGR group (407/3250 (12.5%) and 224/3250 (6.9%), respectively) (*P* < 0.001). Nulliparity was more prevalent in the FGR group (149/250 (59.6%)) compared with the non‐FGR group (1560/3250 (48.0%)), and among parous cases there was a higher incidence of previous SGA (*P* < 0.001). Smoking was also more common in the FGR group compared with the non‐FGR group (14/250 (5.6%) *vs* 57/3250 (1.8%); *P* < 0.001). The median birth‐weight percentile in the most recent previous pregnancy was lower in the FGR group compared with the non‐FGR group (12.0 (IQR, 3.6–32.3) *vs* 43.7 (IQR, 18.3–69.0); *P* < 0.001). For parous women, the median interpregnancy interval was greater in the FGR group compared with the non‐FGR group (3.66 (IQR, 1.88–6.24) years *vs* 2.48 (IQR, 1.57–4.19) years; *P* = 0.002). Chronic hypertension was more common in the FGR group compared with the non‐FGR group (8/250 (3.2%) *vs* 22/3250 (0.7%); *P* < 0.001). Finally, a higher incidence of systemic lupus erythematosus (SLE) and/or antiphospholipid syndrome (APS) was observed in the FGR group compared with the non‐FGR group (4/250 (1.6%) *vs* 11/3250 (0.3%); *P* = 0.018).

There were statistically significant differences between the FGR and non‐FGR groups for all the biomarkers evaluated (Table 1). Of note, the median FTPV *Z*‐score was –0.52 (IQR, –1.01 to 0.06) in the FGR group, compared with –0.01 (IQR, –0.06 to 0.61) in the non‐FGR group (*P* < 0.001).

### Assessment of multicollinearity

**Figure 2:**
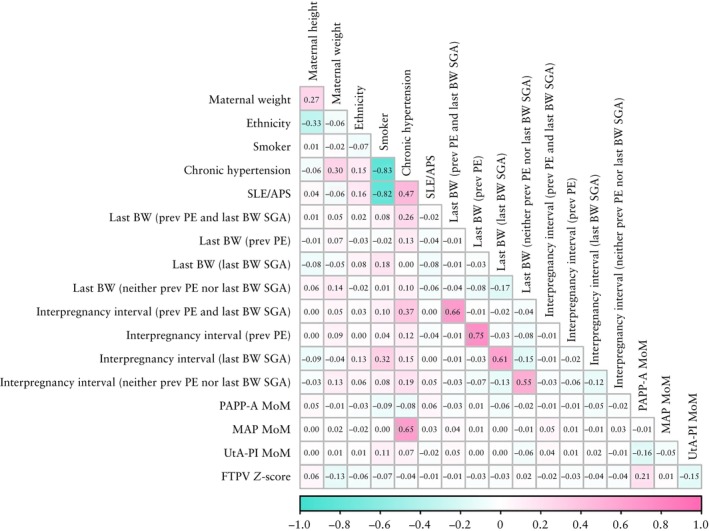
Correlation matrix of factors associated with fetal growth restriction. Values are correlation coefficient (*r*). APS, antiphospholipid syndrome; BW, birth weight; FTPV, first‐trimester placental volume; MAP, mean arterial pressure; MoM, multiples of the median; PAPP‐A, pregnancy‐associated plasma protein‐A; PE, pre‐eclampsia; prev, previous; SGA, small‐for‐gestational age; SLE, systemic lupus erythematosus; UtA‐PI, uterine artery pulsatility index.

The correlation matrix is presented in Figure 2. There was no substantial correlation between most variable pairs. A strong correlation (|r| > 0.8) was observed between smoking and chronic hypertension, and between smoking and SLE/APS. While an association between chronic hypertension and smoking is to be expected, the association between smoking and SLE/APS may be an artifact due to the small number of cases. Importantly, FTPV *Z*‐score was not correlated strongly with any other variable.

### Prediction of FGR by FTPV

Crude and adjusted ORs for FGR are presented in Table 2. FTPV yielded a crude OR of 0.576 (95% CI, 0.496–0.667) for FGR per unit increase in *Z*‐score (or in other terms, the OR for FGR per unit decrease in FTPV *Z*‐score was 1.736 (95% CI, 1.499–2.015)), and this remained statistically significant after adjustment for maternal factors alone, or maternal factors plus biomarkers.

**Table 2:** Crude and adjusted odds ratios (OR) for fetal growth restriction per unit increase or decrease in first‐trimester placental volume (FTPV) *Z*‐score

Variable	FTPV*	Maternal factors + FTPV†	Maternal factors + biomarkers + FTPV‡
FTPV *Z*‐score (per 1‐unit increase)	0.576 (0.496–0.667)	0.570 (0.486–0.666)	0.662 (0.561–0.779)
FTPV *Z*‐score (per 1‐unit decrease)	1.736 (1.499–2.015)	1.756 (1.502–2.059)	1.510 (1.283–1.783)

Values in parentheses are 95% CI.

* Crude OR from univariable analysis.

† Adjusted OR from multivariable analysis including maternal weight, height, ethnicity, smoking status, chronic hypertension and systemic lupus erythematosus/antiphospholipid syndrome; previous pre‐eclampsia (PE), small‐for‐gestational‐age (SGA) birth weight (BW) in last pregnancy, previous PE plus last BW SGA, and neither previous PE nor last BW SGA are included as interaction terms, in combination with both interpregnancy interval and last BW percentile (Model 1b).

‡ Adjusted OR from multivariable analysis including maternal factors plus pregnancy‐associated plasma protein‐A multiples of the median (MoM), mean arterial pressure MoM and uterine artery pulsatility index MoM (Model 3b).

### Evaluation of relationship between FTPV and FGR

**Figure 3:**
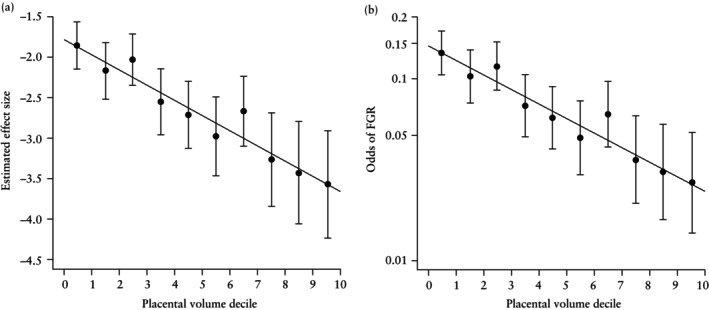
Estimated effect size (a) and odds (logarithmic scale) (b) of fetal growth restriction (FGR) according to first‐trimester placental volume decile. Error bars indicate 95% CI.

**Figure 4:**
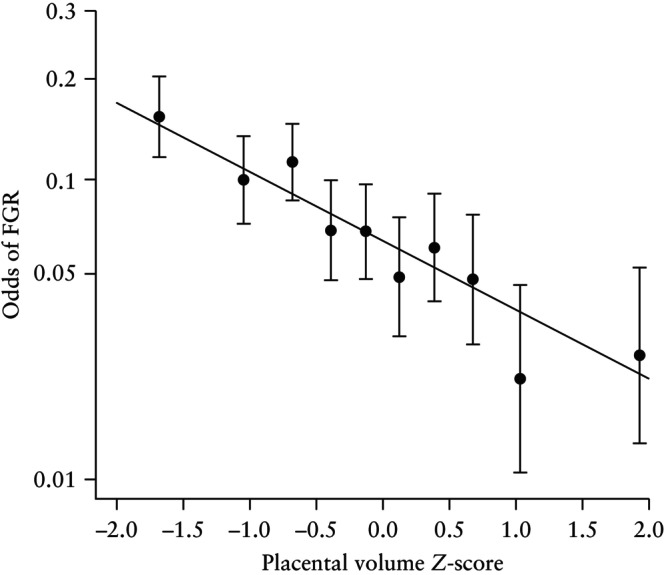
Odds of fetal growth restriction (FGR) (logarithmic scale) according to first‐trimester placental volume *Z*‐score. Data are plotted at mean *Z‐*score for each decile of placental volume. Error bars are 95% CI.

Visualization of model estimates demonstrated consistent decreases in effect size and odds of FGR across FTPV deciles (Figure 3), highlighting both the relative strength of association across deciles and the linear trend. Fitted probabilities plotted on the logit probability scale reinforced the linear trend (Figure 4), supporting the use of placental volume as a continuous variable without transformation.

Additionally, comparison of extreme percentiles (< 10^th^ *vs* > 90^th^) revealed a markedly higher FGR risk in the lowest decile compared with the highest decile, with an estimated OR of 4.670 (95% CI, 3.158–6.906).

### Comparison of models

Table 3 presents the AUC values for the models developed for predicting all cases of FGR. For the prediction of all FGR, without FTPV, the model based on maternal factors and biomarkers demonstrated the best performance, achieving an AUC of 0.78 (95% CI, 0.75–0.81). Incorporating FTPV into this model increased the AUC to 0.79 (95% CI, 0.76–0.82), which was statistically significant (*P* = 0.005).

**Table 3:** Model performance for prediction of all cases of fetal growth restriction

		Sensitivity (95% CI)				
Model	Model components	5% FPR	10% FPR	20% FPR	AUC (95% CI)	*P*
1a	Maternal factors	0.20 (0.15–0.25)	0.27 (0.20–0.34)	0.44 (0.37–0.50)	0.72 (0.70–0.75)	< 0.001
1b	Maternal factors + FTPV	0.23 (0.16 –0.29)	0.37 (0.32–0.43)	0.57 (0.50–0.64)	0.76 (0.73–0.79)	
2a	Biomarkers	0.20 (0.14–0.25)	0.30 (0.24–0.36)	0.46 (0.39–0.52)	0.68 (0.65–0.72)	0.003
2b	Biomarkers + FTPV	0.22 (0.17–0.28)	0.31 (0.24–0.37)	0.50 (0.44–0.56)	0.71 (0.67–0.74)	
3a	Maternal factors + biomarkers	0.26 (0.20–0.32)	0.40 (0.34–0.47)	0.59 (0.51–0.66)	0.78 (0.75–0.81)	0.005
3b	Maternal factors + biomarkers + FTPV	0.29 (0.22–0.35)	0.42 (0.36–0.48)	0.62 (0.55–0.68)	0.79 (0.76–0.82)	

AUC, area under receiver‐operating‐characteristics curve; FPR, false‐positive rate; FTPV, first‐trimester placental volume.

**Figure 5:**
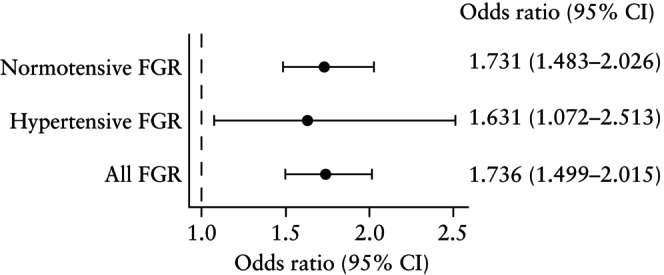
Forest plot showing crude odds ratios for normotensive fetal growth restriction (FGR), hypertensive FGR and all FGR per unit decrease in first‐trimester placental volume *Z*‐score.

Similar results were observed for the models developed to predict normotensive FGR specifically (Table S2), where the addition of FTPV to maternal factors and biomarkers significantly increased the AUC from 0.77 (95% CI, 0.74–0.80) to 0.78 (95% CI, 0.75–0.81), which was statistically significant (*P* = 0.01). ORs derived using univariable logistic regression for normotensive FGR, hypertensive FGR and all FGR are compared in Figure 5. FTPV was associated significantly with FGR in both subgroups, with ORs per unit decrease in FTPV *Z‐*score of 1.731 (95% CI, 1.483–2.026) for normotensive FGR and 1.631 (95% CI, 1.072–2.513) for hypertensive FGR.

The models for the prediction of preterm FGR demonstrated little improvement with the addition of FTPV, and the small differences observed were not statistically significant (Table S3). Of note, the low event rate for preterm FGR (*n* = 39) means that this study is underpowered for this outcome.

**Figure 6:**
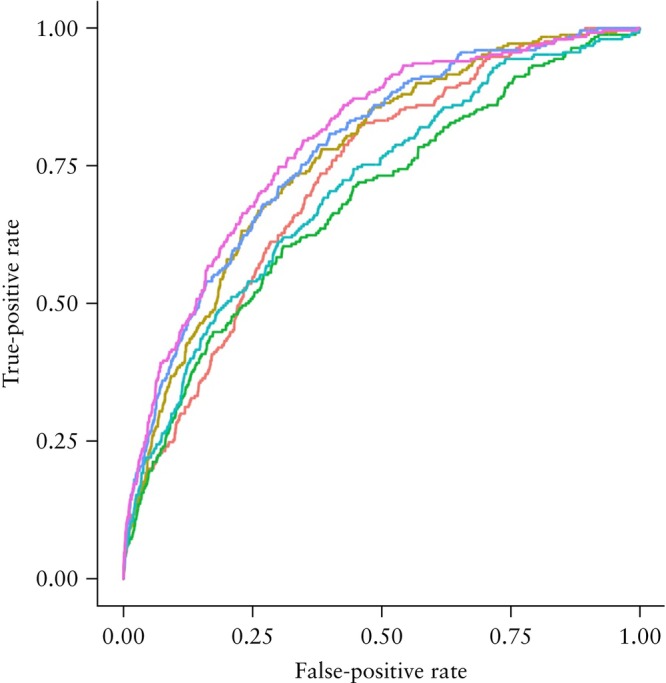
Receiver‐operating‐characteristics curves for models to predict fetal growth restriction. 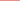, maternal factors (Model 1a); 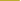, maternal factors + first‐trimester placental volume (FTPV) (Model 1b); 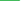, biomarkers (Model 2a); 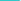, biomarkers + FTPV (Model 2b); 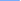, maternal factors + biomarkers (Model 3a); 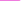, maternal factors + biomarkers + FTPV (Model 3b).

ROC curves for the prediction of all FGR, normotensive FGR and preterm FGR are presented in Figures 6, S1 and S2, respectively.

## DISCUSSION

Our study has shown that integrating OxNNet‐derived FTPV into multivariable predictive models for FGR significantly enhances discriminative ability across all cases of FGR, and this remained true for the subgroup of normotensive FGR. This finding is valuable, as it indicates that low placental volume is predictive of FGR specifically, and not merely predictive of hypertensive disorders, which are correlated strongly with FGR. Little improvement was seen in the prediction of FGR with delivery < 37 weeks; however, the study was underpowered to achieve statistical significance owing to the low rate of preterm FGR in this cohort. An alternative approach to the prediction of preterm FGR would be to construct survival models with FGR (or specifically the birth of a baby with FGR) treated as an event in time, and this is an area for future exploration.

Our findings align with previous research. For instance, Plasencia *et al*.^29^ conducted a study involving 3103 singleton pregnancies, in which FTPV was measured between 11 and 13 weeks. Birth weight < 5^th^ percentile (SGA) was used as an indicator of suboptimal fetal growth. The study found that incorporating placental volume into predictive models enhanced the predictive performance for SGA compared with using maternal characteristics alone. Multivariate regression analysis indicated that placental volume provided a significant independent contribution to the prediction of both SGA (OR, 0.10 (95% CI, 0.02–0.41); *P* = 0.001) and large‐for‐gestational age (OR, 12.92 (95% CI, 2.59–64.59); *P* = 0.002) when added to maternal characteristics and serum PAPP‐A. However, the inclusion of placental volume did not substantially improve the overall screening performance for SGA achieved using maternal characteristics and serum PAPP‐A (AUC, 0.692 (95% CI, 0.645–0.738) *vs* 0.706 (95% CI, 0.660–0.753); *P* = 0.160).

González‐González *et al*.^30^ conducted a study involving 1004 singleton pregnancies to develop predictive models for identifying fetuses with a birth weight < 10^th^ percentile, using customized reference charts. The most successful model in the first trimester included maternal characteristics, UtA‐PI and placental volume as predictors. This model achieved an AUC of 0.735 (95% CI, 0.696–0.773), demonstrating moderate discriminative ability. Importantly, the inclusion of placental volume alongside UtA‐PI and maternal characteristics highlights the value of integrating placental assessment into early‐pregnancy screening protocols.

In comparison, our study benefitted from a larger sample size, which enhanced the precision of our modeling and strengthened the robustness of our findings. Additionally, the broader scope of our cohort ensured improved representation of minority groups and better generalizability of our findings across diverse populations. Furthermore, our model for the prediction of FGR using FTPV, maternal factors and first‐trimester biomarkers (Model 3b) outperforms those published previously with respect to AUC values, although it is important to note that external validation of the model was beyond the scope of this project.

A notable strength of our study is the integration of the OxNNet toolkit. This advanced AI‐based tool enables fully automated placental segmentation and volume calculation, which allowed us to compile a larger dataset than would have been achievable using the traditional gold standard of manual segmentation. Furthermore, the OxNNet toolkit offers a clear advantage over the Virtual Organ Computer‐aided Analysis (VOCAL™) method used by both Plasencia *et al*. and González‐González *et al*., which depends on geometric assumptions that may not accurately capture the irregular shape of the placenta, particularly during the first trimester^29^, ^30^, ^31^. Research has shown significant variability in placental shape in early pregnancy, which could lead to inaccuracies in volume estimation when using manual or geometry‐based approaches^32^. By addressing these limitations, the OxNNet toolkit enhances both the accuracy and efficiency of placental volume measurement. Our study was also strengthened by the implementation of rigorous quality‐control measures for both placental imaging and segmentation. These stringent protocols ensured the accuracy and reliability of placental volume assessments used in our models, improving the robustness and credibility of our findings.

In this study, the definition of FGR differed from those of Plasencia *et al*.^29^ and González‐González *et al*.^30^, who used birth weight < 5^th^ percentile on population‐based charts and < 10^th^ percentile on customized charts, respectively. We adopted a stricter threshold of < 3^rd^ percentile to ensure that identified cases represent true FGR, rather than constitutional smallness. However, reliance on this threshold alone may have excluded some true FGR cases with birth weight ≥ 3^rd^ percentile. To address this limitation, we also included cases with a birth weight < 10^th^ percentile when abnormal Doppler indices were detected antenatally, where constitutional smallness is unlikely. This combined approach provided a more robust and comprehensive definition of FGR compared with previous studies. Nonetheless, this definition may encompass instances of FGR not attributable to placental causes, although the exclusion of fetal chromosomal and significant structural abnormalities helped to mitigate this concern.

The established relationship between birth weight and placental weight at birth creates a potential for collinearity when including placental volume in predictive models for FGR. This is because, even early in gestation, low placental volume may already be a proxy marker of FGR. However, FTPV *Z*‐score is derived relative to CRL, thereby serving not as an absolute measure of placental size but as an index of deviation from expected placental development for a given fetal size. This distinction is critical: it transforms FTPV into a biologically meaningful marker of disproportionate placental growth, which we have shown to be associated strongly with an increased risk for FGR. Thus, the inclusion of FTPV *Z*‐score enhanced the prediction of FGR rather than confounding the models. We also found no strong associations between FTPV *Z*‐score and other model components, suggesting that multicollinearity is unlikely to compromise model stability.

A limitation of this study is its reliance on simple regression models and the absence of more rigorous internal validation strategies. While our approach aligns well with the stated aims of this paper, the development of models intended for clinical application would benefit from internal validation techniques, such as bootstrapping or cross‐validation, to enhance robustness and generalizability.

A further limitation is that our models were not adjusted for aspirin use. There is evidence to suggest that low‐dose aspirin is preventative of FGR^33^, and thus, the predictive power of the models and individual markers may be underestimated in this study. This does not affect the validity of our conclusions but is an important consideration in the proposed future development of definitive models incorporating FTPV for the prediction of FGR. These models will require robust external validation on a larger dataset, and will require exploration of calibration metrics, clinical decision thresholds and decision‐curve analysis in order to establish their clinical utility.

In summary, this study demonstrates that FTPV percentile is a predictor of FGR and underscores the value of incorporating FTPV in predictive models for FGR. The development of the OxNNet toolkit for the automated measurement of placental volume represents a significant advance in first‐trimester placental ultrasonography and opens the possibility for the use of FTPV in population‐level screening, contingent upon further refinement and external validation of the predictive models. Our findings support the integration of advanced placental assessment into early‐pregnancy screening protocols, with the potential to improve the identification and management of FGR.

## Supporting information


**Table S1** Ultrasound machine settings.

**Table S2** Model performance for prediction of normotensive fetal growth restriction.

**Table S3** Model performance for prediction of preterm fetal growth restriction.

**Appendix S1** Summary of models to predict fetal growth restriction.

**Appendix S2** RData file containing models to predict fetal growth restriction.

**Figure S1** Receiver‐operating‐characteristics curves for models to predict normotensive fetal growth restriction.

**Figure S2** Receiver‐operating‐characteristics curves for models to predict preterm fetal growth restriction.

## Data Availability

The data that support the findings of this study are available on request from the corresponding author. The data are not publicly available due to privacy or ethical restrictions.
