# Supplementary material for: First‐trimester Placental Ultrasound (FirstPLUS) study: prediction of fetal growth restriction using OxNNet‐derived first‐trimester placental volume
Source: Ultrasound Obstet Gynecol. 2025 Dec 6;67(1):49–59. doi: 10.1002/uog.70146 (PMC12757825; doi:10.1002/uog.70146)
Supplement: Supplementary file 1 — Table S1 Ultrasound machine settings. [file UOG-67-49-s006.docx]

**Table S1:** Ultrasound machine settings

| **Menu** | **Parameter** | **Setting** |
| --- | --- | --- |
| Main 2D | XBeam Compound receive imaging | Off |
|  | Speckle reduction imaging | 3 |
|  | Gray map | 16 |
|  | Tint Map | Off |
|  | Angle | 90° |
|  | Time gain compensation | Far Field |
| Sub 2D | Gray map | 16 |
|  | Tint | Off |
|  | Line filter | High |
|  | Persistence | 6 |
|  | Enhance | 2 |
|  | Line density | Normal |
|  | Reject | 20 |
|  | Output thermal index | Normal |
| 3D | Volume angle | 85° |
|  | Quality | High1 |
| Power Doppler | Quality | *Norm* |
|  | WMF | *Low 1* |
|  | PRF | *0.9KHz* |
| Sub power Doppler | PD Map | *5* |
|  | Frequency | *Low* |
|  | Flow Res | *Mid 2* |
|  | L Filter | *2* |
|  | Artefact | *On* |
|  | Smooth | *Rise 4* |
|  | Line Dens | *6* |
|  | Smooth | *Fall 5* |
|  | Ensemble | *15* |
|  | Balance | *150* |
