# Supplementary material for: First‐trimester Placental Ultrasound (FirstPLUS) study: prediction of fetal growth restriction using OxNNet‐derived first‐trimester placental volume
Source: Ultrasound Obstet Gynecol. 2025 Dec 6;67(1):49–59. doi: 10.1002/uog.70146 (PMC12757825; doi:10.1002/uog.70146)
Supplement: Supplementary file 2 — Table S2 Model performance for prediction of normotensive fetal growth restriction. [file UOG-67-49-s002.docx]

**Table S2:** Model performance for prediction of normotensive fetal growth restriction

| **Model** | **Components** | **Sensitivity (95% CI)** | | | **AUC (95% CI)** | ***P* value** |
| --- | --- | --- | --- | --- | --- | --- |
|  |  | **5% FPR** | **10%FPR** | **20% FPR** |  |  |
| 7a | Maternal factors | 0.19  (0.14–0.25) | 0.25  (0.19–0.32) | 0.47  (0.39–0.53) | 0.71  (0.68–0.75) | <0.001 |
| 7b | Maternal factors + FTPV | 0.21  (0.15–0.27) | 0.34  (0.28–0.41) | 0.52  (0.46–0.60) | 0.75  (0.72–0.78) |  |
| 8a | Biomarkers | 0.15  (0.10–0.20) | 0.25  (0.19–0.31) | 0.43  (0.36–0.49) | 0.67  (0.63–0.71) | 0.006 |
| 8b | Biomarkers + FTPV | 0.17  (0.13–0.23) | 0.27  (0.21–0.35) | 0.45  (0.38–0.52) | 0.70  (0.66–0.73) |  |
| 9a | Maternal factors + biomarkers | 0.23  (0.17–0.29) | 0.35  (0.29–0.44) | 0.55  (0.47–0.61) | 0.77  (0.74–0.80) | 0.01 |
| 9b | Maternal factors + biomarkers + FTPV | 0.26  (0.20–0.33) | 0.39  (0.33–0.47) | 0.58  (0.51–0.65) | 0.78  (0.75–0.81) |  |

**AUC:** Area under receiver operating characteristic curve

**CI:** Confidence interval

**FPR:** False positive rate

**FTPV:** First trimester placental volume
