# Supplementary material for: First‐trimester Placental Ultrasound (FirstPLUS) study: prediction of fetal growth restriction using OxNNet‐derived first‐trimester placental volume
Source: Ultrasound Obstet Gynecol. 2025 Dec 6;67(1):49–59. doi: 10.1002/uog.70146 (PMC12757825; doi:10.1002/uog.70146)
Supplement: Supplementary file 3 — Table S3 Model performance for prediction of preterm fetal growth restriction. [file UOG-67-49-s001.docx]

**Table S3:** Model performance for prediction of preterm fetal growth restriction

| **Model** | **Model components** | **Sensitivity (95% CI)** | | | **AUC (95% CI)** | ***P* value** |
| --- | --- | --- | --- | --- | --- | --- |
|  |  | **5% FPR** | **10%FPR** | **20% FPR** |  |  |
| 4a | Maternal factors | 0.30  (0.18–0.46) | 0.36  (0.23–0.54) | 0.49  (0.33–0.64) | 0.74  (0.66–0.82) | 0.1 |
| 4b | Maternal factors + FTPV | 0.33  (0.18–0.49) | 0.43  (0.28–0.58) | 0.54  (0.38–0.69) | 0.78  (0.71–0.85) |  |
| 5a | Biomarkers | 0.38  (0.23–0.53) | 0.56  (0.41–0.72) | 0.67  (0.51–0.79) | 0.79  (0.71–0.87) | 0.91 |
| 5b | Biomarkers + FTPV | 0.41  (0.26–0.56) | 0.62  (0.46–0.74) | 0.64  (0.49–0.79) | 0.79  (0.71–0.87) |  |
| 6a | Maternal factors + biomarkers | 0.51  (0.36–0.69) | 0.62  (0.46–0.77) | 0.77  (0.64–0.90) | 0.85  (0.78–0.92) | 0.93 |
| 6b | Maternal factors + biomarkers + FTPV | 0.51  (0.38–0.69) | 0.62  (0.46–0.79) | 0.74  (0.62–0.90) | 0.85  (0.79–0.92) |  |

**AUC:** Area under receiver operating characteristic curve

**CI:** Confidence interval

**FPR:** False positive rate

**FTPV:** First trimester placental volume
