# Supplementary figures and images for: First‐trimester Placental Ultrasound (FirstPLUS) study: prediction of fetal growth restriction using OxNNet‐derived first‐trimester placental volume
Source: Ultrasound Obstet Gynecol. 2025 Dec 6;67(1):49–59. doi: 10.1002/uog.70146 (PMC12757825; doi:10.1002/uog.70146)

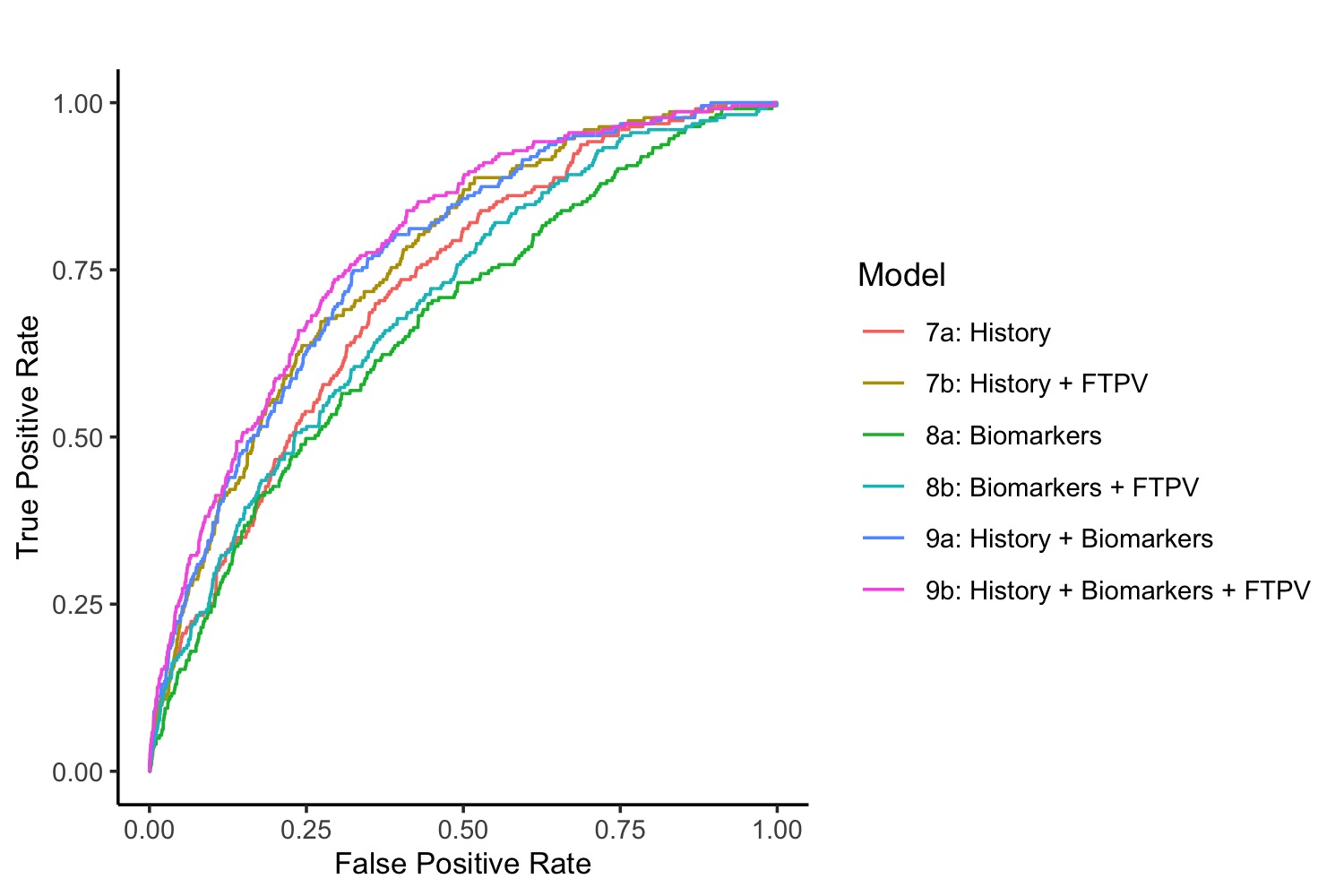

Supplement: Supplementary file 6 — Figure S1 Receiver‐operating‐characteristics curves for models to predict normotensive fetal growth restriction. [file UOG-67-49-s004.jpeg]

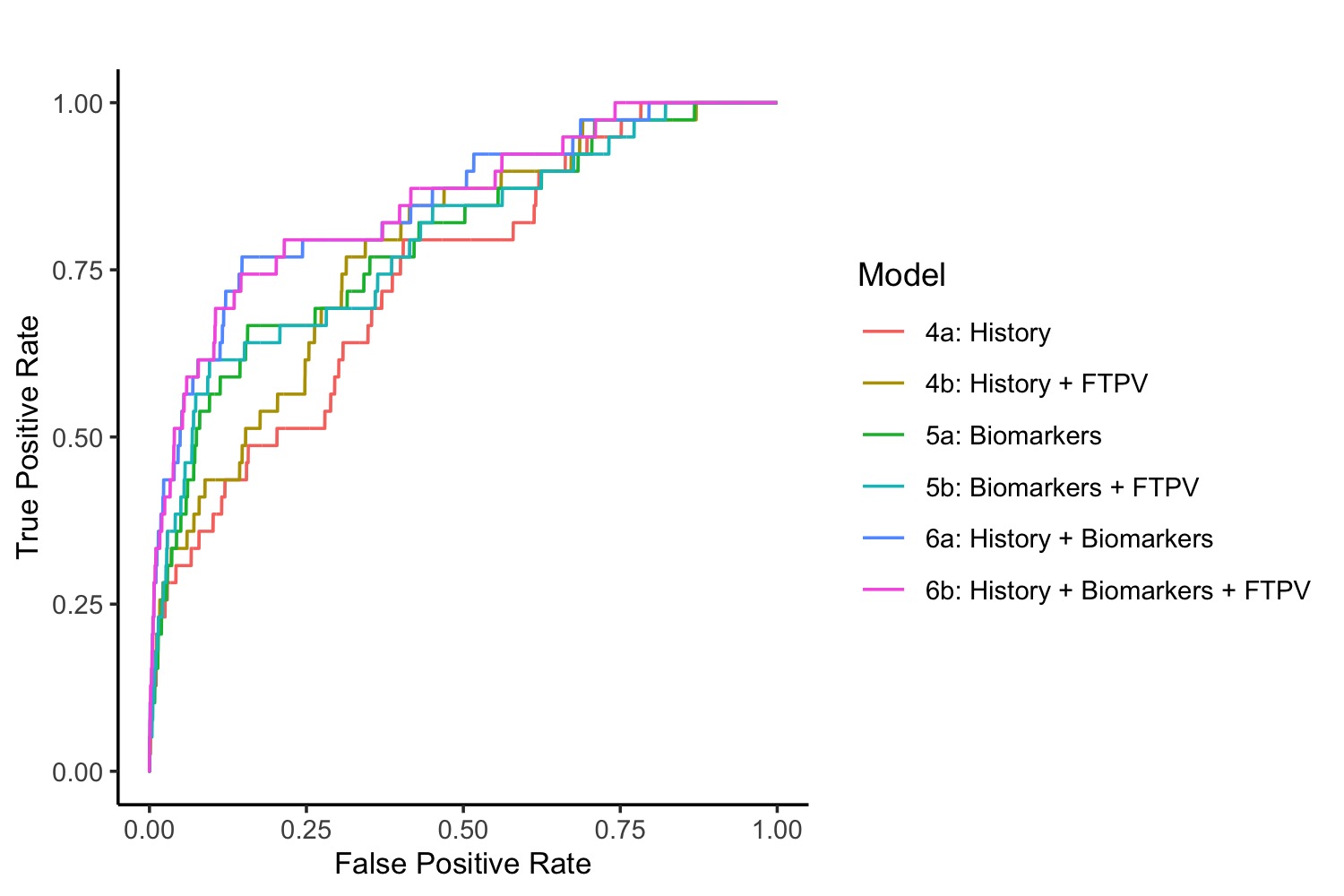

Supplement: Supplementary file 7 — Figure S2 Receiver‐operating‐characteristics curves for models to predict preterm fetal growth restriction. [file UOG-67-49-s007.jpeg]
